# A biophysical study on molecular physiology of the uncoupling proteins of the central nervous system

**DOI:** 10.1042/BSR20150130

**Published:** 2015-07-14

**Authors:** Tuan Hoang, Miljan Kuljanin, Matthew D. Smith, Masoud Jelokhani-Niaraki

**Affiliations:** * Department of Chemistry and Biochemistry, Wilfrid Laurier University, Waterloo, Ontario, Canada, N2L 3C5; † Department of Biology, Wilfrid Laurier University, Waterloo, Ontario, Canada, N2L 3C5; ‡ Biophysics Interdepartmental Group, University of Guelph, Guelph, Ontario, Canada, N1G 2W1

**Keywords:** central nervous system (CNS), circular dichroism (CD) spectroscopy, fatty acid (FA), membrane protein folding, molecular association, proton transport, reconstitution, uncoupling proteins (UCPs)

## Abstract

Mitochondrial inner membrane uncoupling proteins (UCPs) facilitate transmembrane (TM) proton flux and consequently reduce the membrane potential and ATP production. It has been proposed that the three neuronal human UCPs (UCP2, UCP4 and UCP5) in the central nervous system (CNS) play significant roles in reducing cellular oxidative stress. However, the structure and ion transport mechanism of these proteins remain relatively unexplored. Recently, we reported a novel expression system for obtaining functionally folded UCP1 in bacterial membranes and applied this system to obtain highly pure neuronal UCPs in high yields. In the present study, we report on the structure and function of the three neuronal UCP homologues. Reconstituted neuronal UCPs were dominantly helical in lipid membranes and transported protons in the presence of physiologically-relevant fatty acid (FA) activators. Under similar conditions, all neuronal UCPs also exhibited chloride transport activities that were partially inhibited by FAs. CD, fluorescence and MS measurements and semi-native gel electrophoresis collectively suggest that the reconstituted proteins self-associate in the lipid membranes. Based on SDS titration experiments and other evidence, a general molecular model for the monomeric, dimeric and tetrameric functional forms of UCPs in lipid membranes is proposed. In addition to their shared structural and ion transport features, neuronal UCPs differ in their conformations and proton transport activities (and possibly mechanism) in the presence of different FA activators. The differences in FA-activated UCP-mediated proton transport could serve as an essential factor in understanding and differentiating the physiological roles of UCP homologues in the CNS.

## Abstract (precis)

Uncoupling proteins (UCP)2, UCP4 and UCP5 transport protons across the inner membrane of mitochondria in the central nervous system (CNS). Novel recombinant protein expression allowed expression of UCPs in *Escherichia coli* membranes. Functional neuronal UCPs formed multimers in membranes and interacted with various fatty acids (FAs) to transport protons. Self-association and unique ion transport properties of UCPs distinguish their physiological roles in the CNS.

## INTRODUCTION

Located in the inner mitochondrial membrane (IMM), uncoupling proteins (UCPs) dissipate the proton electrochemical gradient across the membrane, resulting in the reduction of ATP production through ATP synthase. Abundantly expressed in brown adipose tissue (BAT), human UCP1 mediates proton transport across the IMM and generates heat [1]. Expressed in the central nervous system (CNS), UCP2, UCP4 and UCP5 are referred to as neuronal UCPs [2]. UCP2 is also found in several other human tissues in addition to the CNS, including liver, endothelium and pancreatic tissues; and might be responsible for different physiological functions [2–4]. UCP4 and UCP5 are predominantly localized in neurons and neurosensory cells and their physiological roles are not clear [2,5–7]. Compared with the prototypic UCP1, the amino acid sequence identities of UCPs 2, 4, 5 are 59%, 34% and 30% respectively [8–11]. Despite these relatively low sequence identities to UCP1, both UCP4 and UCP5 possess comparable helical conformations and proton transport activities to other UCPs [11]. Neuronal UCP-mediated proton transport is activated by fatty acids (FAs) and inhibited by purine nucleotides (PNs) [11]. Recent studies relate the proton transport-mediated uncoupling activities of neuronal UCPs to possible protection against reactive oxygen species (ROS), produced in the mitochondria [4,12–17]. In addition to the proton transport function, all UCPs are also able to transport chloride and other small anions [9,11,18]. Protein sequence symmetry analysis and experimental results suggest that UCPs could also function as small substrate carriers using an H^+^-assisted mechanism [19,20]. The exact physiological role(s) of neuronal UCPs has not been fully established.

Examining the structure-function relationships of neuronal UCPs remains an intriguing approach for clarifying the physiological roles of these proteins in neurons. In our previous studies, using recombinant proteins expressed in bacterial systems, we obtained highly pure neuronal UCPs in inclusion bodies and reconstituted these proteins into mild detergents and liposomes for structural and functional studies [10,11,21]. Although this expression method produces proteins in high yield and allows for excellent purity, there is always a possibility of partial folding or misfolding of the reconstituted proteins that can influence the accurate analysis of their molecular functions. We recently reported a novel recombinant protein expression system using the auto-induction method and the small periplasmic leader sequence PelB (pectate lyase B) to direct UCP1 to the bacterial membrane, which resulted in the production of highly pure native-like folded UCP1, in high yields [22]. In that report, recombinant UCP1 extracted from the bacterial membranes and reconstituted in both detergents and liposomes adopted dominantly helical structures and displayed proton and chloride ion transport activity [22]. In the current study, we applied this novel expression method and report the expression of His-tagged recombinant versions of neuronal UCPs in *Escherichia coli* inner membranes for the first time. All three neuronal UCPs were extracted from bacterial membranes using the mild detergent octyl glucoside (OG) and reconstituted into liposomes. Similar to UCP1 [22], the neuronal UCPs showed evidence of self-association and displayed predominantly helical conformations in liposomes. The associated forms of neuronal UCPs also transported proton and chloride ions across lipid membranes. The effect of various FAs on the overall structure and the proton/chloride transport rates of neu-ronal UCPs led to new insights into specific functions of neuronal UCPs and possible differentiation of their physiological roles.

## MATERIALS AND METHODS

### UCP constructs

The human UCP2 cDNA clone (pET21a-UCP2) was a gift from M. Brand (MRC Dunn Human Nutrition Unit, Cambridge, U.K.). UCP4 cDNA was synthesized by GenScript Corp. and received in a pUC57 vector. UCP5 cDNA was purchased from A.T.C.C. and was received in a pCR-BluntII-TOPO vector. The neuronal UCP constructs were sub-cloned into the pET21d vector (Novagen) and modified to contain an N-terminal His-tag followed by the tobacco etch virus (TEV) protease recognition site (ENLYFQG), as previously described [10]. The His_6_-ENLYFQG-hUCPs coding sequences were sub-cloned into the pET26b(+) vector using standard molecular techniques such that the PelB leader sequence was in frame with the His-tag, TEV protease site and the protein. The constructs (pET26b-hUCP2, pET26b-hUCP4 and pET26b-hUCP5) were then transformed into *E. coli* BL21 CodonPlus (DE3)-RIPL for production of the recombinant fusion proteins containing an N-terminal PelB leader sequence fused to the respective neuronal UCP. To verify the identity of the clones, each of the recombinant cDNAs in the pET26b (+) expression vector was sequenced (TCAG, Sick Children's Hospital).

### Chemicals

Egg yolk L-α-lecithin (Sigma) contained at least 60% (w/w) phosphatidylcholine. The remaining 40% was comprised mostly of phosphatidylethanolamine and other lipids. The phospholipid POPC (1-palmitoyl-2-oleoyl-sn-glycero-3-phosphocholine) was from Avanti Polar Lipids (Alabaster). C_8_E_4_ (octyltetraoxyethylene) was obtained from Bachem. DDM was from Calbiochem-EMD Biosciences. 3-[(3-cholamidopropyl)dimethylammonio]-1-propanesulfonate (CHAPS) and OG were from Affymetrix. The fluorescent probe SPQ [6-methoxy-N-(3-sulfopropyl)quinolinium; 99% purity] was from Biotium Inc.. All other chemicals were from Sigma.

### Expression, membrane extraction and purification of neuronal UCPs

Recombinant versions of neuronal UCPs were overexpressed in *E. coli* BL21 CodonPlus (DE3)-RIPL using the auto-induction method, as previously described for UCP1 [22]. Briefly, the bacterial culture was grown in the auto-induction media [1% tryptone, 0.5% yeast extract, 1 mM MgSO_4_, 0.5% glycerol, 0.05% glucose, 0.2% lactose, 25 mM (NH_4_)_2_SO_4_, 50 mM KH_2_PO_4_, 50 mM Na_2_HPO_4_] at 22°C for 20 h before collection [23]. The bacterial cell pellets were collected at 8000 ***g*** for 10 min (4°C). Bacterial cells were suspended in extraction buffer (20 mM Tris/HCl, 500 mM NaCl, pH 8.0) and lysed using a Constant System cell disruption press (20 kPsi) in the presence of complete protease inhibitor cocktail (EDTA-free; Roche), DNase (0.5 mg/ml) and lysozyme (0.2 mg/ml). The cell lysate was subjected to centrifugation at 20000 ***g*** for 20 min (4°C) to remove inclusion bodies and unbroken cells. The supernatant was subsequently ultracentrifuged at 256631 ***g*** (TLA-100 rotor, Beckman Coulter) for 1 h (4°C) to yield the bacterial membranes in the pellet fraction and the cytosol in the supernatant. The enrichment of membranes in the pellet fractions was confirmed using the NADH oxidase assay [22].

The purification protocols of UCP2, UCP4 and UCP5 followed that of UCP1, as described previously [22]. In brief, the bacterial membranes were solubilized in binding buffer {20 mM Tris/HCl, 500 mM NaCl, 1mM [Tris (hydroxypropyl) phosphine], pH 8.0} containing 1% LDAO (lauryldimethylamine-oxide) detergent and 20 mM imidazole. This mixture was subjected to Ni-NTA (nitrilotriacetic acid) chromatography for purification under non-denaturing conditions. The columns were washed with 30 mM imidazole and eluted with 400 mM imidazole in 1% OG detergent. The pooled eluted fractions were desalted using Econo-Pac 10DG Columns (Bio-Rad). The final purity and concentration of purified proteins (∼0.3–0.5 mg protein/1 l culture) were analysed by SDS/PAGE and Bradford protein concentration assays respectively. The proteins were stored in desalting buffer (1% OG, 50 mM NaCl, 20 mM Tris/HCl, 1% glycerol, pH 8.0) at −80°C.

### Semi-native PAGE

To provide semi-native conditions, the content of SDS was significantly reduced compared with traditional SDS/PAGE [22]. Following the protocol from our previous study, 2 mM SDS was included in the electrophoresis running buffer to ensure the migration of proteins. Prior to electrophoretic experiments, each protein sample was mixed with the sample buffer containing 62.5 mM Tris/HCl, pH 6.8, 0.002% Bromophenol Blue and 10% glycerol. The gels were run at 110 V for 75 min and stained with Coomassie Brilliant Blue R-250.

### Identification of proteins by western blots and MS analysis

The identity of overexpressed His_6_-tagged UCP2, UCP4 and UCP5 was confirmed by both western blot and MS analyses. In the western blot, 10–50 μg of total proteins were run on a SDS/PAGE (12% gel) and transferred (110 min, 15 V) to nitrocellulose membranes using the semi-dry technique. The membranes were stained with Amido Black to confirm the efficiency of transfer and were blocked overnight at 4°C in TBS-buffer containing 5% skim milk and 0.05% Tween-20. Mouse IgG2b anti-histidine antibody (Millipore) was used as the primary antibody (1:500 dilution). Rabbit peroxidase-conjugated antibody raised against mouse (Rockland) was used as the secondary antibody (1:4000 dilution). Immunodetection was achieved by chemiluminescence using ECL Western Blot reagent (GE Healthcare) and an image was captured using a Bio-Rad VersaDoc Imaging System.

To further confirm the identity of recombinant protein in bands of different molecular masses, purified protein bands resolved on electrophoretic gels were excised, trypsin-digested and analysed by LC-MS/MS in a data-dependent acquisition, as described previously [22]. Trypsin-digested protein fractions were separated using a NanoAcquity (Waters) LC-MS/MS equipped with a 25-cm × 75-μm inner diameter C18 column. MS analysis was performed on a QExactive (Thermo Scientific) using higher energy collision dissociation. *De novo* sequencing of fractionated proteins was performed with Peak Studio 6.0 Software using the Uniprot Swiss database for *Homo sapiens*, with a false discovery rate of <1%.

### Reconstitution of UCPs into liposomes

Reconstitution of UCPs into liposomes followed the procedures in previously used protocols with minor modifications [11,22]. Briefly, stock lipids in chloroform were dried under vacuum (8-12 h) and resuspended in reconstitution buffers. The multilamellar liposomes were solubilized in C_8_E_4_ detergent [(detergent/lipid=2.5/1 (w/w)]. In the ion transport assays, the internal reconstitution buffer was supplemented with 3 mM fluorescent probe SPQ. Purified proteins were added to the transparent lipid/detergent mixed micelles. The spontaneous formation of liposomes/proteoliposomes was achieved following the detergent removal by SM-2 Biobeads (Bio-Rad). In ion transport assays, the external probe SPQ was removed using a coarse Sephadex G25-300 (GE Healthcare) spin column.

### CD and fluorescence spectroscopic measurements

Far- and near- UV CD spectra were measured in 0.1 and 0.5 cm path length quartz cells respectively, on an Aviv 215 spectropolarimeter (Aviv Biomedical) at 1-nm resolution. Proteins’ ellipticities are exhibited as mean residue ellipiticity [θ] and the reported spectra are an average of at least eight measurements. Secondary structure content of proteins was estimated using the deconvolution program CDSSTR and the analysis was based on a set of 48 reference proteins and performed on the Dichroweb web site [24,25]. Thermal denaturation of neuronal UCPs in detergents was analysed by monitoring the decrease in protein ellipiticity at 222 nm, in the range of 15°C–90°C.

Steady-state fluorescence measurements were performed using a Cary Eclipse spectrophotometer (Varian). Excitation and emission bandwidth slits for all measurements were 5 nm and a scan speed of 600 nm/min was used throughout the experiments (25°C).

### Ion transport measurements

Proton and chloride transport measurements were performed as previously described [11,22]. Following reconstitution, liposomes/proteoliposomes were formed with entrapped SPQ fluorescent probes. SPQ is quenched by anions. In each ion transport assay, 40 μl of liposomes/proteoliposomes were incubated in 1.96 ml of external buffer. In the proton transport assays, the internal medium consisted of TES buffer (20 mM), TEA_2_SO_4_ (TEA, tetraethylammonium; 54 mM) and EDTA (0.7 mM). The external medium in this case contained K_2_SO_4_ (54 mM) instead of TEA_2_SO_4_. In the chloride transport assays, the internal medium consisted of 110 mM sodium phosphate and 0.2 mM EDTA, whereas the external buffer contained 10 mM sodium phosphate 200 mM KCl and 0.2 mM EDTA. All media were kept constant at pH 7.2. The ion fluxes in both assays were initiated by the influx of K^+^ from the external media mediated by the K^+^-ionophore valinomycin. In the chloride transport assays, chloride influx quenched the SPQ and caused a decrease in its fluorescence signal. In the proton transport assays, FAs activated UCP-mediated proton efflux, resulting in deprotonation of TES buffer. TES anion quenched SPQ's fluorescence. All ion transport data were corrected by subtraction of the non-specific ion leakage and calibrated for the SPQ fluorescence response and internal volume of proteoliposomes. The final protein concentrations in proteoliposomes were quantified using a modified Lowry concentration assay, as described previously [11].

### Sequence alignment and structural modelling of neuronal UCPs

Protein primary sequence alignment of UCPs and ADP/ATP carrier (AAC) were performed using Clustal Omega [26]. Protein sequence alignment analysis was viewed through Jalview program [27]. Protein helical contents were estimated by Distill program [28]. The 3D structural model of UCP2 (PDB ID: 2LCK) was viewed using Pymol (http://www.pymol.org) [30].

## RESULTS

### Expression of neuronal UCPs in bacterial membranes and their folding in detergents and liposomes

The incorporation of the PelB leader sequence into the N-terminus of the neuronal UCPs combined with the application of the auto-induction method resulted in all three neuronal UCPs (UCP2, UCP4 and UCP5) being targeted to the bacterial membranes. Bacterial cells were fractionated using differential velocity centrifugation and the identity of the neuronal UCPs in the *E. coli* membrane fraction was verified using Western blot analysis probed with anti-histidine antibody (Figure 1, inset) and mass spectrometric analysis (Supplementary Table S1). An NADH oxidase activity assay was used to confirm that the membrane fraction containing the UCPs was in fact enriched in bacterial membranes (results not shown).

**Figure 1:**
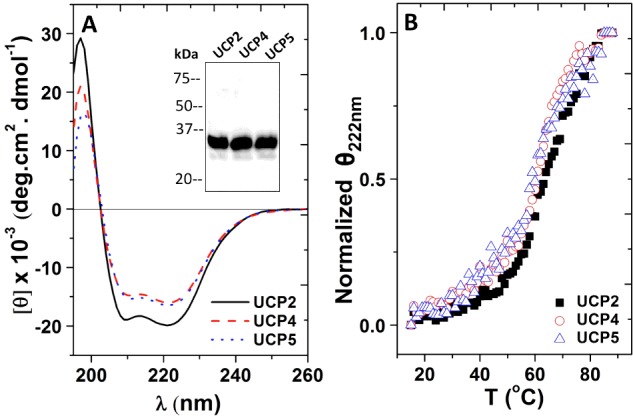
Expression of neuronal UCPs in bacterial membranes and folding of the proteins in the detergent OG (**A**) Averaged far-UV CD spectra of 3–5 μM neuronal UCPs in buffer containing 1% OG. Inset shows the western blot analysis of neuronal UCPs in the membrane fraction isolated from *E. coli* probed with mouse IgG2b anti-histidine antibody, detected by chemiluminescence using a horseradish peroxidase-linked secondary antibody. Molecular mass markers (kDa) are indicated to the left. (**B**) Comparative thermal denaturation of neuronal UCPs in 1% OG. The molar ellipticity of UCPs at 222 nm was normalized and *T*_m_ for each UCP was calculated using Hill equation to fit the data

Neuronal UCPs were extracted from the bacterial membranes using the mild detergent OG and purified using nickel affinity chromatography, prior to being reconstituted into liposomes. The folding of neuronal UCPs in OG was examined using CD spectroscopy (Figure 1A; Table 1). As shown in the far-UV CD spectra, neuronal UCPs in 1% OG exhibited typical spectral footprints of helical conformations, with double negative maximum ellipticities at ∼222 and ∼208 nm and a positive maximum ellipticity of ∼195 nm (Figure 1A). Deconvolution of these CD spectra revealed 63%, 53% and 54% helical contents for UCP2, UCP4 and UCP5 respectively (Table 1). These helical contents were significantly higher than those obtained previously using recombinant UCPs refolded from inclusion bodies [11]. It is also important to note that the estimated helicities of neuronal UCPs extracted from the membranes are close to the protein helicities calculated based on the primary amino acid sequences (Table 1). To further characterize the stability of neuronal UCPs in OG, thermal denaturation experiments were performed in which the protein ellipticity at 222 nm was monitored in the 15°C–90°C temperature range (Figure 1B). All three proteins displayed relatively stable conformations that underwent a two-state unfolding process, with melting points in the range of 64°C–69°C (Figure 1B; Table 2).

**Table 1:** Estimated secondary structure composition of neuronal UCPs in detergents*, in lipid vesicles* and from primary amino acid sequence prediction^†^

Protein	Protein environment	α-Helix	β-Strand	Turn	Random	NRMSD
	OG micelles	63	5	12	19	0.014
UCP2	POPC vesicles	85	3	2	8	0.002
	Distill prediction	69	–	–	–	–
	OG micelles	53	7	16	24	0.015
UCP4	POPC vesicles	80	6	4	11	0.009
	Distill prediction	70	–	–	–	–
	OG micelles	54	9	17	20	0.009
UCP5	POPC vesicles	85	4	3	7	0.009
	Distill prediction	75	–	–	–	–

*Deconvolution of CD spectra was performed using the CDSSTR program on the Dichroweb website (see ’Materials and Methods’). The values represent the percentage of secondary structure composition.NRMSD, normalized RMSD, denotes the best fit between the calculated and experimental CD spectra.

†Helical contents of neuronal UCPs were predicted using the Distill prediction program, as described in the ‘Materials and Methods’.

**Table 2:** Stability of neuronal UCPs in the OG detergent and liposomes

		Dissociation constants: SDS titration of reconstituted UCPs* (mM SDS)			
Protein	OG detergent *T*_m_^†^	CD *K*_1/2_	Fl *K*_a1/2_	Fl *K*_b1/2_	Δ*λ*_max_
UCP2	69.3±1.1	1.1±0.1	0.61±0.1	7.1±0.3	5–7
UCP4	64.5±1.9	1.0±0.1	0.60±0.1	9.8±0.3	7–8
UCP5	67.7±2.6	1.0±0.1	0.60±0.1	8.9±0.2	7–8

†*T*_m_ (melting temperature) of UCPs in the detergent OG was calculated using the Hill equation to fit the data of the thermal denaturation curve depicted in Figure 1(B).

*Dissociation constants were calculated using the Hill equation to fit the data of SDS dissociation experiments for reconstituted UCPs in liposomes. The CD *K*_1/2_ values were obtained from fitting the θ_208_/θ_222_ compared with [SDS] curve, which represented the SDS concentrations at 50% of protein dissociation (Figures 2A–2F). The Fl *K*_a1/2_ values were obtained from fitting the maximum Trp fluorescence intensity of UCPs compared with [SDS] at a low SDS concentration range (0–10 mM; Figures 3A–3F). The Fl *K*_b1/2_ values were obtained from fitting the maximum Trp fluorescence intensity of UCPs compared with [SDS] at a high SDS concentration range (10–100 mM; Figures 3A–3F). The Δ*λ*_max_ indicates the range of SDS concentrations where Trp's *λ*_max_ started to red-shift (Figures 3G–3I).

Abbreviation: Fl, fluorescence.

### Self-association of neuronal UCPs in liposomes

Following isolation in detergent, the recombinant neuronal UCPs were reconstituted into liposomes to allow for characterization in a more native-like membrane environment. CD spectra of UCPs in liposomes displayed characteristics typical of α-helices, with the π ➔ π* exciton split bands at ∼192 and 208 nm and the n ➔ π* transition at ∼222 nm (Figures 2A–2C). Compared with their conformation in detergent, each of the neuronal UCPs in liposomes displayed a marked enhancement in their helical content, which could indicate the important role of lipids in the folding of these proteins. Despite their high helical content, the neuronal UCPs exhibited atypical far-UV CD spectra for helical proteins. Specifically, the parallel π ➔ π* band of neuronal UCPs' far-UV CD spectra at 208–210 nm exhibited a shoulder-like negative maximum with lower ellipticity than the negative maximum at 222 nm (Figure 2). The θ_208_/θ_222_ ratios of the CD spectra, in both OG detergent and liposomes, were less than 1 (0.60, 0.63 and 0.61 for UCP2, UCP4 and UCP5 respectively). Such ellipticity ratios have been observed in the spectra of coiled coil and helical bundle motifs and as a result of protein association [22,31–34]. Using complementary electrophoresis and MS methods, the atypical helical conformation of the neuronal UCPs can mostly probably be attributed to a predominant population of UCP multimers (tetramers) in liposomes with molecular mass of ∼ 140–150 kDa (Figures 2G–2I; Supplementary Table S1). This is the first direct observation for the self-association of neuronal UCPs *in vitro*. A similar phenomenon was reported previously for UCP1 [22].

**Figure 2:**
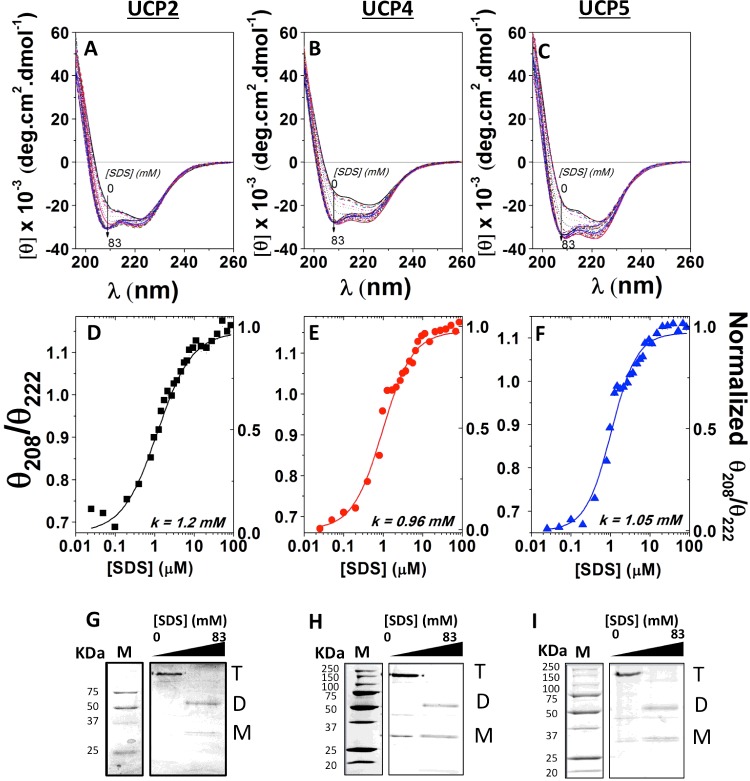
Titration of neuronal UCPs in POPC liposomes with SDS, monitored by CD spectroscopy and semi-native PAGE (**A**, **B**) and **C**) show the far-UV CD spectra of reconstituted UCP2, UCP4 and UCP5 respectively in POPC liposomes titrated with SDS (0–83 mM). Corresponding plot of θ_208_/θ_222_ compared with [SDS] reveals a cooperative dissociation of UCP2 (**D**), UCP4 (**E**) and UCP5 (**F**) tetramers in the presence of SDS. The θ_208_/θ_222_ ratio was normalized and *K*_1/2_ ([SDS] at 50% protein dissociation) was calculated using the Hill fitting. The *K*_1/2_ values of UCP2, UCP4 and UCP5 are 1.2 mM SDS, 0.96 mM SDS and 1.05 mM SDS respectively. The corresponding semi-native PAGE gels (12%) show reconstituted UCP2 (**G**), UCP4 (**H**) and UCP5 (**I**) in POPC liposomes titrated with SDS, stained with Coomassie Brilliant Blue. UCP tetramers (***T***) were the predominant species detected in POPC liposomes when treated with loading buffer lacking SDS. As the SDS concentration increased beyond the critical micelle concentration of SDS (∼2 mM), transition towards predominantly UCP dimers (***D***) was observed. At 83 mM SDS (∼40× CMC of SDS), UCP tetramers were completely absent, UCP dimers became prominent and a small concentration of monomers (***M***) was present.

### SDS titration of associated neuronal UCPs

To characterize the stability of the associated forms of neuronal UCPs, the proteoliposomes were titrated with SDS (Figures 2A–2F and 3). It was expected that with increasing SDS concentration a gradual dissociation of the multimers would occur until their complete dissociation at high SDS concentrations [22]. During SDS titrations, overall and local conformational changes of the proteins were monitored by CD and fluorescence spectroscopies respectively (Figures 2A–2F and 3). Figures 2(A)–2(C) show the far-UV CD spectra of the titrations of reconstituted neuronal UCP tetramers with SDS. The θ_208_/θ_222_ ratios in the spectra of each protein were plotted against SDS concentrations to monitor the gradual transformation of the proteins from their associated to monomeric forms (Figures 2D–2F). The far-UV CD spectra of reconstituted UCPs displayed characteristics of associated conformations, with a negative maximum at 222 nm and a negative shoulder at ∼210 nm (θ_208_/θ_222_ < 1; Figures 2A–2C). As the SDS concentration increased from 0 to 2 mM, the CD spectral shape of UCPs exhibited a drastic change. The negative ellipticities at 210 and 222 nm were both enhanced and the negative shoulder at 210 blue-shifted towards 208 nm and gradually became a distinct minimum (Figures 2A–2C). Concurrent with this transformation, the θ_208_/θ_222_ ratios increased and approached the value of 1 (Figures 2D–2F). These results suggest a transition of reconstituted UCPs towards a less constrained multimeric conformation with weaker intermolecular interactions and more independent monomers. Addition of SDS above 2 mM (2–83 mM) did not affect the θ_208_ ellipticity, whereas the θ_222_ negative ellipticity further decreased (Figures 2A–2C). Above 50 mM SDS, the θ_208_/θ_222_ was greater than 1 and remained unchanged, suggesting a stable conformation of dissociated proteins (Figures 2D–2F). As discussed in a previous study, high concentrations of SDS could also induce the denaturation of protein tertiary structures [22]. Plotting the θ_208_/θ_222_ ratio as a function of [SDS] (mM) revealed a two-state dissociation path for neuronal UCPs reconstituted in POPC liposomes, with the dissociation constant *K*_1/2_ representing the SDS concentration at which 50% of the protein population was dissociated (Figures 2D–2F). The *K*_1/2_ values of UCP2, UCP4 and UCP5 were determined to be approximately 1 mM SDS (Table 2). Semi-native PAGE analysis of UCPs complemented the CD results, showing that addition of SDS to proteoliposomes resulted in the gradual loss of tetramers and concurrent enrichment of dimers and monomers (Figures 2G–2I). At the end titration point of 83 mM SDS, the UCP tetramers were completely replaced by UCP dimers and monomers (Figures 2G–2I; Supplementary Table S1).

Dissociation/unfolding of UCPs by SDS was also monitored using fluorescence spectroscopy (Figures 3A–3C). The total Trp fluorescence of all neuronal UCPs (*λ*_ex_=295 nm) increased and the maximum emission (*λ*_max_) gradually red-shifted in the presence of increasing concentrations of SDS (Figures 3A–3C). Plotting the fluorescence intensity compared with [SDS] revealed a three-state transition curve for each of the neuronal UCPs (Figures 3D–3F). Signs of this three-state dissociation/transition were also apparent (but less resolved) in the ellipticity ratio compared with [SDS] plots of UCPs 4 and 5 (and less for UCP2; Figures 2D–2F). The first transition for all neuronal UCPs occurred at an SDS concentration of ∼0.6 mM, which aligned with the conformational dissociation point (∼1 mM) observed using CD (Figures 2D–2E). The second transition occurred at higher SDS concentrations (∼7–10 mM), which could be interpreted as the point at which further dissociation of UCP dimers to monomers and partial unfolding of monomers occurs (Figures 3D–3F; Table 2). Examination of the *λ*_max_ of Trp compared with [SDS] revealed that Trp residues experienced a red shift towards higher wavelengths (335–339 nm) at SDS concentrations between ∼5–8 mM, signifying the exposure of Trp residues to more polar environments (Figures 3G–3I). This shift in *λ*_max_ of Trp residues occurred at [SDS] close to the second transition point observed for UCP fluorescence intensities (Figures 3D–3F; Table 2).

**Figure 3:**
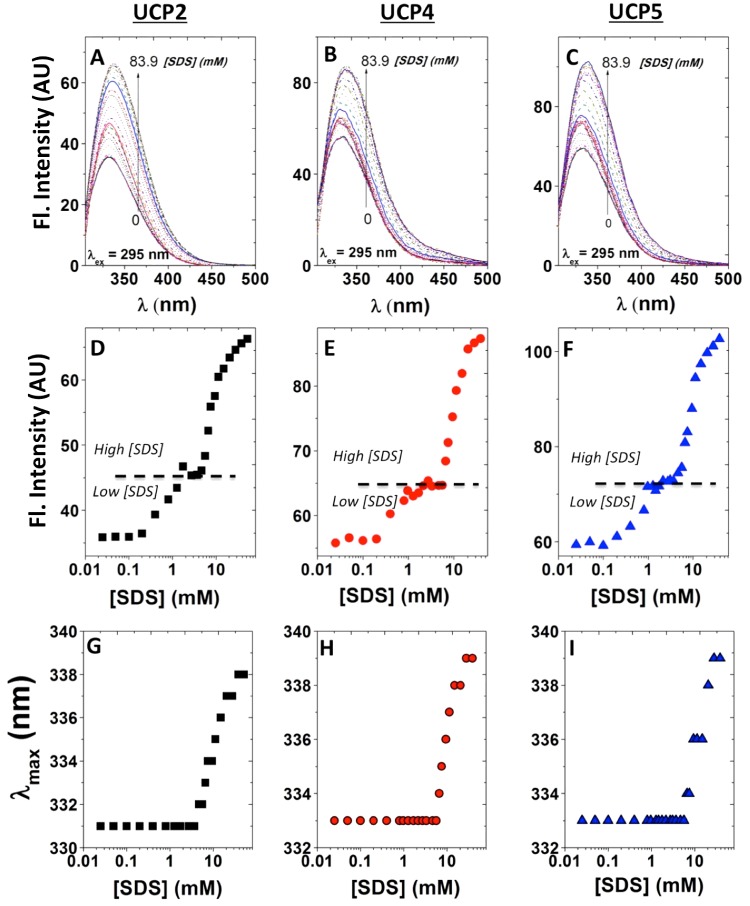
Dissociation of neuronal UCPs in POPC liposomes by SDS, monitored by Trp fluorescence The Trp fluorescence spectra of UCP2 (**A**), UCP4 (**B**) and UCP5 (**C**) (*λ*_ex_=295 nm) were monitored as SDS was added to the proteoliposomes ([SDS]=0–83.9 mM). The corresponding plots of maximum Trp intensity reveal a three-state dissociation process for neuronal UCP tetramers in lipid vesicles, as shown for UCP2 (**D**), UCP4 (**E**) and UCP5 (**F**). In the low [SDS] region (0–10 mM), UCP tetramers are suggested to transition toward tight dimeric units. In the high [SDS] region (10–100 mM), UCP tight dimers could be further dissociated into their loose dimeric and monomeric forms. The *K*_1/2_ for the first (*K*_a1/2_) and second (*K*_b1/2_) transitions of all neuronal UCPs were obtained and summarized in Table 2. In addition, corresponding plots of the maximum emission wavelength (*λ*_max_) compared with [SDS] for UCP2 (**G**), UCP4 (**H**) and UCP5 (**I**) reveal no significant change in the maximum Trp emission wavelength up to ∼10 mM SDS, above which the fluorophores became more exposed to the polar environments and exhibited a red shift in its maximum emission wavelength.

In summary, the results from CD and fluorescence spectroscopy measurements, semi-native PAGE and MS suggest that neuronal UCPs are able to self-associate into tetramers in lipid membranes under our experimental conditions. These results also show two distinct transition states for neuronal UCP tetramer dissociation during SDS titration experiments. The first transition occurs at low SDS concentrations (∼1 mM), where UCP tetramers are dissociated into their corresponding dimeric units. Conformational analysis of neuronal UCPs revealed a transition from highly associated to less-constrained helices (Figure 2). At the low SDS concentration range, Trp residues remain in relatively less polar environments, as shown by the *λ*_max_ values of ∼331–333 nm (Figure 3). The second transition occurred at higher SDS concentrations (∼10 mM), where neuronal UCP dimers were further dissociated into a mixture of loose dimers and monomers. During this transition, neuronal UCPs exhibited small overall conformational changes and their Trp residues were exposed to more polar environments (*λ*_max_ ∼ 338–339 nm).

### Effects of FAs on the proton and chloride transports mediated by neuronal UCPs

In an effort to characterize the FA-activation mechanism of the UCP-mediated proton transport pathway, we assessed the effects of various FAs on UCP-mediated ion transport rates (Figures 4 and 5). In addition, CD spectroscopy was used to examine the FA–UCP interactions (Supplementary Figures S1 and S2). In the present study, seven different FAs were used, which varied in their hydrophobicity, structure and hydrocarbon chain length lauric acid (LA), palmitic acid (PA), oleic acid (OA), linoleic acid (LLA), linolenic acid (LNA), arachidonic acid (AA) and docohexaneoic acid (DHA; Figure 4B).

**Figure 4:**
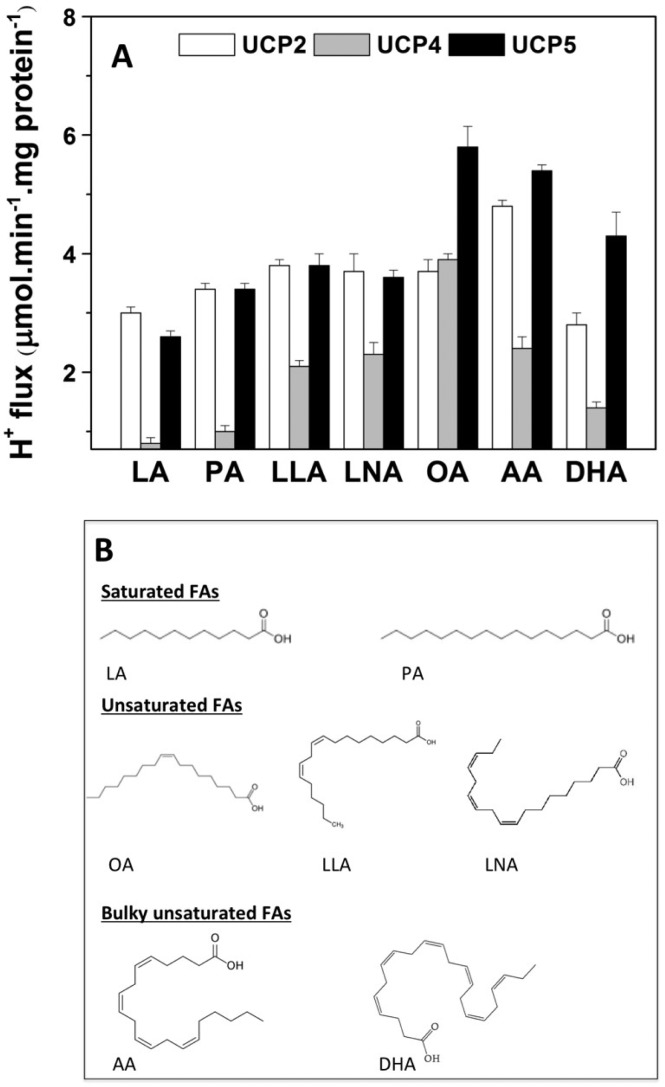
Effect of FAs on the proton transport activity of associated neuronal UCPs in liposomes (**A**) Average corrected proton transport rates mediated by neuronal UCPs in L-α-lecithin vesicles. FAs were used at the concentration of 50 μM to activate the proton transport by UCPs. The reported rates are the means of 5–10 independent measurements±the S.E.M.s. The phospholipid concentration was ∼20 mg/ml; the final protein content in the liposomes was 2–5 μg/mg of lipid. The final proton transport rates were corrected for the total protein content in the liposomes. (**B**) Structure of FAs tested in the present study.

Results from CD spectroscopy and proton transport experiments showed that all FAs interacted with UCPs and differentially activated their proton transport activities (Figure 4; Supplementary Figures S1 and S2). The UCP-mediated proton transport rates varied between 1–6 μmol·min^−1^·mg protein^−1^ [or 0.5–3 protons per second for each UCP protein], depending on the specific UCP and FA (Figure 4). A comparative proton transport rate in all neuronal UCPs shows that with the exception of DHA, unsaturated FAs are generally stronger proton transport activators for neuronal UCPs than saturated FAs (LA and PA; Figure 4). As revealed by far- and near-UV CD spectra, minor conformational changes in UCPs imply that, among the tested FAs, LA had the weakest interaction with these proteins (Supplementary Figures S1 and S2). In general, based on the enhanced negative double maxima ellipticities at the 208 and 222 nm wavelengths in the far-UV CD spectra, it is apparent that FA–UCP interactions induce less densely packed protein conformations (Supplementary Figure S1). Near-UV CD spectra of neuronal UCPs also show an enhanced overall signal intensity of UCPs in the presence of FAs (except for LA; Supplementary Figure S2). Among neuronal UCPs, UCP4 exhibited the lowest proton transport rates (except in the case of OA activation; Figure 4). In the presence of the same FA activator, UCP4-mediated proton transport rates were ∼2–3 fold lower than those of UCP2 and UCP5 (Figure 4). However, in the presence of OA, UCP4 exhibited its highest proton transport rate at ∼4 μmol·min^−1^·mg protein^−1^, similar to that of UCP2 (Figure 4). OA was also the strongest proton transport activator for UCP5 (Figure 4). On the other hand, AA was the most potent proton transport activator for UCP2 (Figure 4). Using planar lipid bilayer systems, it was also shown that AA is a strong FA activator for the proton transport activity of UCP2 [35].

In addition to proton transport, chloride transport was also detected for reconstituted neuronal UCPs (Figure 5). Overall, all neuronal UCPs transported chloride ions at lower rates than protons (Figures 4 and 5). UCP2 transported chlorides at a higher rate (∼1 μmol·min^−1^·mg·protein^−1^) compared with UCP4 and UCP5 (∼ 0.5 μmol·min^−1^·mg·protein^−1^; Figure 5). To further examine the ion transport mechanism of UCPs, we also examined the effects of FAs (PA and AA) on the chloride transport activity of neuronal UCPs in proteoliposomes. At the concentration of 50 μM, both PA and AA partially inhibited chloride transport by all neuronal UCPs (Figure 5). AA, a more potent UCP-proton transport activator, displayed a slightly stronger inhibitory effect on the chloride transport of all UCPs (Figures 4 and 5). Consequently, our experimental results suggest that FAs interact with UCPs and differentially modulate their proton (activated) and chloride (inhibited/reduced) transport activities.

**Figure 5:**
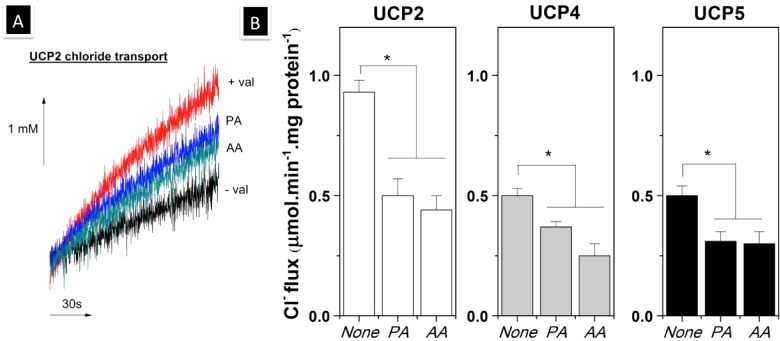
Effect of FAs on the chloride transport activity of associated neuronal UCPs in liposomes (**A**) Chloride influx mediated by UCP2 in the absence (+val) and presence of PA (PA) or AA (AA). The non-UCP2 specific flux was shown for comparison (−val). (**B**) Average corrected chloride transport rates mediated by neuronal UCPs in L-α-lecithin vesicles. FAs were used at the concentration of 50 μM to examine their effect on the chloride transport by UCPs. The reported rates are the means of 5–10 independent measurements±the S.E.M.s. The phospholipid concentration was ∼20 mg/ml; the final protein content in the liposomes was 2–5 μg/mg of lipid. The final chloride transport rates were corrected for the total protein content in the liposomes. A one-way ANOVA statistical test was performed to determine the statistical significance of data, and *P*-values were obtained. *P*<0.05 when comparing the chloride transport mediated by neuronal UCPs in the absence and presence of FAs (*).

## DISCUSSION

### Novel recombinant expression for studying the structure and function of neuronal UCPs

Molecular and structural studies of UCPs have been challenging due to difficulties in obtaining sufficient yield of functional proteins reconstituted in lipid membranes [36]. While facilitating the production of large quantities of pure proteins, bacterial expression systems also pose challenges for expression of heterologous proteins, especially membrane proteins such as UCPs [9–11,36,37]. Previous expression of mammalian UCPs in *E. coli* resulted in the production of protein inclusion bodies [10,11]. Once extracted from inclusion bodies and reconstituted in model membranes, the proteins might be only partially folded into their functional forms [10,11]. To improve the folding quality of the neuronal UCPs, we utilized the PelB leader sequence to target the proteins to bacterial membranes, as we have done previously for UCP1 [22]. This novel expression system proved to be successful for neuronal UCPs as well (Figure 1A, inset). The neuronal UCPs that were targeted to the bacterial membranes were folded, highly helical and stable in both detergents and liposomes (Figures 1 and 2; Table 1). In addition, all reconstituted neuronal UCPs displayed putative UCP ion transport functions (Figures 4 and 5). It is important to note that UCP5, when folded from inclusion bodies in a previous study, exhibited chloride transport in L-α-PC (phosphatidylcholine) liposomes only when cardiolipin (CL) was included with the membrane lipid [11]. In the current study, reconstituted UCP5 transported chloride in L-α-PC liposomes in the absence of added CL (Figure 5). Based on our previous studies, the presence of CL can enhance the ion transport activity of UCP5 as well as other UCPs [11,22]. Given that *E. coli* membranes contain ∼5% CL, UCP5 localized to the bacterial membranes could associate with CL and the intact UCP5–CL complex could be maintained and carried into liposomes during the purification and reconstitution processes [38]. It is also possible that UCP5 extracted from bacterial membranes possesses a different conformation from that of UCP5 extracted from inclusion bodies and therefore facilitated chloride transport. Consequently, the fusion of the PelB leader sequence, together with the use of mild auto-induction expression method, allows for the preparation of pure neuronal UCPs in high yield that are suitable for structural and functional studies.

### Understanding the self-association of neuronal UCPs in lipid membranes

Self-association of mitochondrial carrier family (MCF) proteins has been reported previously in several studies [22,39–41]. As with our previous study on UCP1 [22], the predominant molecular form of neuronal UCPs in liposomes, observed in the current study, was tetrameric (Figure 1A, inset; Supplementary Table S1). Tetrameric UCPs were capable of transporting both protons and chloride ions across lipid bilayers (Figures 4 and 5). The effect of SDS titration on the tetrameric forms of neuronal UCPs, as revealed by their electrophoretic and mass spectrometric analyses, further confirms our previously proposed molecular model for UCP1 multimerization (Figure 6) [22]. In this molecular model, neuronal UCP tetramers can be easily dissociated into loosely-bound dimeric units at low SDS concentrations (0.6–1 mM; Figures 2, 3 and 6). Within each UCP dimer, monomers interact strongly and the dimeric unit cannot be completely dissociated to monomers even at a high concentration of SDS (∼83 mM; Figure 6). Other studies in the past also reported the existence of UCP1 dimers [39,42] and the dimeric unit of UCPs (UCPs 1, 2, 4 and 5) could be physiologically significant [22]. The dimerization of other mitochondrial carriers has been reported to be important for the metabolite transport function of these proteins [43–45]. In our previous study, neuronal UCPs transported protons and chloride in their monomeric forms [11]. The results of the current study imply that UCPs can also transport protons (FA-activated) in multimeric forms.

**Figure 6:**
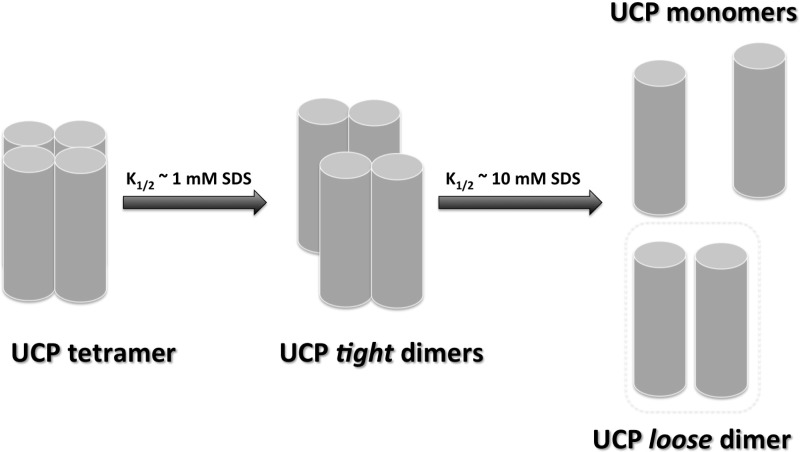
Dissociation model of neuronal UCP tetramers Neuronal UCP tetramers are composed of two loosely associated homo-dimers. The tetramers can be dissociated into homo-dimers at low SDS concentrations ([SDS] ∼ CMC). UCP tetramers were dissociated at ∼1 mM SDS to form dimers. UCP monomers interact and tightly associate with each other through direct protein–protein interactions and/or protein interactions mediated by lipids, to form a tight dimer. These tight dimers can be partially unfolded/dissociated at higher concentrations of SDS to form loose dimers and/or monomers. Loose dimers may be fully separated to monomers at much higher concentrations of SDS.

The self-association of neuronal UCPs and other UCP homologues can occur via protein–protein interaction or protein—lipid–protein interaction. Amino acid sequence analysis revealed the existence of conserved GxxxG motifs in all UCPs [46]. These motifs are commonly found in TM (transmembrane) domains that are involved in helix–helix associations; their presence in the TM domains of UCPs provides indirect support for the likelihood of UCP self-association, discussed above (Table 3) [46]. In addition, sequence alignment of UCPs demonstrated many conserved amino acid sequences that could participate in protein self-association (Table 3). In our previous study of UCP2 mutants, two positively charged residues located in TM2 domain at the protein/IMS (intermembrane space) interface (Arg^96^ and Lys^104^) were shown to participate in UCP2 protein–protein interactions [47]. These amino acids are conserved and involved in formation of a salt-bridge network of UCPs in the protein/IMS interface, which have been suggested to play an important role in regulating the substrate-binding and transport of UCPs (Supplementary Figure S3) [19]. In addition to protein–protein interaction, UCP association may also occur through protein–lipid–protein interaction. In our previous study, the mitochondrial lipid CL was shown to influence UCP1 self-association and enhancing its proton transport activity [22]. Therefore, given its abundance in the brain mitochondrial membranes, CL could play an essential role in the molecular association and ion transport activity of neuronal UCPs.

**Table 3:** Similarities and differences in expression profiles, amino acid sequences and common motifs of neuronal UCPs*

Conserved/semi-conserved sequences			
	UCP2	UCP4	UCP5
MCF conserved sequences [48]	Px[D/E]xx[K/R]x[R/K]-20-30 aa-[D/E]Gxxxx[W/Y/F]-[K/R]G		
Inter-helical association motif [46]	GxxxG		
Proton transport residue [54]	Asp^28^	Glu^34^	Glu^55^
Charged residues in chloride-binding region [55]	Asp^35^, Lys^38^, Arg^88^, Asp^138^, Lys^141^, Lys^239^ and Asp^236^	Asp^41^, Lys^44^, Arg^97^, Asp^149^, Lys^152^, Lys^253^ and Asp^250^	Asp^60^, Lys^66^, Arg^113^, Asp^163^, Lys^166^, Lys^260^ and Asp^257^
PN-binding residues [9]	Arg^88^, Arg^185^, Arg^279^	Arg^97^, Arg^199^, Arg^299^	Arg^113^, Arg^206^, Arg^305^
Unique biochemical/biophysical properties			
	UCP2	UCP4	UCP5
Expression profile	Expression in various tissues and organs (brain, muscle, liver, heart, kidney, pancreas)	Primarily expressed in the brain and the CNS (neurons, astrocytes, Purkinje cells)	Primarily expressed in the brain and to a lesser extent in testis, uterus, kidney, lung, stomach
Sequence identity to UCP1	59%	34%	30%
Proton transport rate (μmol·min^−1^·mg·protein^−1^)	∼2–5 (strongest activator: AA)	∼1–4 (strongest activator: OA)	∼2–6 (strongest activator: OA)
Chloride transport rate (μmol·min^−1^·mg·protein^−1^)	∼1	∼0.5	∼0.5
Interaction with CL [11]	CL induced higher UCP2-mediated H^+^ flux	CL had no effect on UCP4-mediated H^+^ flux	CL induced higher UCP5-mediated H^+^ flux

*Conserved/semi-conserved amino acid sequences were extracted from the multiple sequence alignment of neuronal UCPs with other UCPs and AACs. The amino acid sequence alignment of AACs and human UCP homologues were done using the Clustal Omega [26] program and viewed with Jalview [27].

### FA binding and ion transport of neuronal UCPs

Information about the structure and physiological function of brain-specific UCP4 and UCP5 is scarce as compared with other UCPs. With the goal of demystifying the biological function(s) of UCPs in the CNS and characterizing their molecular physiology using a biophysical approach, the current study reports new findings on the structures and ion transport activities of neuronal UCPs, especially UCPs 4 and 5. The data underscore the common structural and functional features of all neuronal UCPs (Table 3). On the other hand, each UCP exhibits variations in its ion (proton and chloride) transport and FA-binding properties, which could be relevant to the specific physiological roles of these proteins in the CNS (Table 3).

All three neuronal UCPs display self-associated helical conformations that transport proton (FA-activated) and chloride (FA-inhibited) ions across lipid membranes (Figures 1–5; Table 3). As members of the MCF, neuronal UCPs share a conserved motif Px[D/E]xx[K/R]x[R/K]-20–30 aa-[D/E]Gxxxx[W/Y/F]-[K/R]G spanning from the odd-numbered TMs (1, 3 and 5) to the even-numbered TMs (2, 4 and 6) [4,8,48]. This signature motif may play an important role in forming general funnel-shaped channel-like structures spanning the inner mitochondrial membranes (Table 3) [30,48]. In addition, neuronal UCPs share UCP-specific conserved amino acids, including the salt-bridge networks at the IMS/protein and mitochondrial matrix/protein interfaces, the GxxxG motifs involved in inter-helical association, the conserved amino acid residues in proton and chloride transport and the conserved arginine residues in PN-binding sites (Table 3). Consequently, the conserved amino acid sequences and motifs in UCPs can translate to their similarity in self-association and ion transport function.

Despite similarity in their general structures and functions, neuronal UCPs displayed distinct proton and chloride transport activities in the presence of various FA activators. For instance, UCP2 transported protons at the highest rate in the presence of AA, whereas UCP4 and UCP5 achieved their maximum proton transport rates in the presence of OA (Figure 4; Table 3). These differences could be related to the specificity in binding of FAs to UCPs. Whereas a specific site for FA–UCP interaction has not been identified, the results from the present study can shed light on understanding the differences in FA-binding regions of neuronal UCPs. Since FAs with longer hydrophobic chains (C16 and above) activated the proton transport of neuronal UCPs more strongly than shorter chain FAs (C12 and below), it is plausible that specific FA-binding regions, with a threshold hydrophobicity, exists in UCPs. These FA-binding hydrophobic regions might also possess a specific geometry that can better accommodate certain shapes of FAs over the others; for example, long chain compared with short chain or bulky compared with linear FAs. It has been also proposed that stable hydrophobic binding between FAs and UCPs is required for proton transport function of the proteins [49]. Therefore, long chain FAs could stay associated with UCPs due to the specific hydrophobic interactions above the threshold hydrophobicity and might also act as a shuttle to translocate protons from the IMM to the mitochondrial matrix. In contrast, in the case of short chain FAs, hydrophobic interactions with UCPs are close to or below threshold hydrophobicity, resulting in weak interactions that lead to FA anions being dissociated from UCPs before becoming protonated. In addition to the possibility of the existence of hydrophobic regions with threshold hydrophobicity, the geometry of FAs might also play a role in their specific interaction with UCPs. For example, with one double bond, the C18-chain OA has a high conformational flexibility (Figure 4). On the other hand, the C20-chain AA possesses a much lower conformational flexibility and a more bulky (and compact) shape due to the presence of four, configurationally restricted, conjugated double bonds (Figure 4). Therefore, the FA-binding hydrophobic region of UCP2 could be broader than those of UCPs 4 and 5 to better accommodate bulky, long-chain FAs, such as AA, rather than more linear, long-chain FAs, such as OA. Overall, in addition to the hydrophobic nature and specific geometry of the FA-binding sites, the configuration of FAs can also influence the specificity of FA binding to UCPs.

### Neuronal UCPs: ion transport mechanism and specific physiological roles

At the moment, two main questions remain unanswered regarding the proton transport mechanism of UCPs and their physiological functions in the mitochondria. Our data suggest that the specific hydrophobic interaction between FAs and UCPs plays an important role in the proton flux mediated by the proteins. As detected through far- and near-UV CD spectra, all FAs interacted with UCPs and induced conformational changes in the proteins. Moreover, compared with short chain FAs, long chain FAs (≥ C16) induced drastic conformational changes and higher proton transport rates in neuronal UCPs (Figures S1 and S2). Similar results were obtained for proton transport in a patch-clamp study of UCP1 in BAT mitochondria [49]. In comparison with their proton transport activity, all neuronal UCPs transported chloride at much lower rates. Interestingly, the chloride transport by all neuronal UCPs was partially inhibited by FAs such as PA and AA. Therefore, FAs could have dual roles in modulating the function of UCPs, as an activator of proton transport and as an inhibitor of chloride transport. It is also possible that both transport pathways might share one or more key amino acids. Identifying these amino acid residues could be essential for elucidating the ion transport mechanism of UCPs, in general, and their proton transport mechanism in particular.

The proton transport function of UCPs in the CNS reduces the electrochemical potential across the IMM, which in turn leads to the reduction of ROS production [4,12–14,50]. However, the question regarding the mechanism of UCP activation to transport protons is not fully answered. As discussed earlier, long chain FAs and their oxidized derivatives can interact with UCPs through hydrophobic interactions and activate proton transport [16,47,49]. A hypothesis for the activation mechanism of proton transport in UCPs suggests the oxidative stress-induced lipolysis pathway [4]. Specifically, under oxidative stress, lipolysis (via mitochondrial phospholipase A_2_) in the mitochondrial membranes can release free FAs (and their oxidized derivatives). These substrates then interact with neuronal UCPs and activate their proton transport pathway in a feedback mechanism (Figure 7) [51,52]. In other words, oxidative stress generates ROS products and free FAs and their oxidized forms as substrates for binding to UCPs and activating their proton transport function. Proton transport in turn leads to the reduction of ROS production and lipolytic activity of phospholipase A_2_. Once a lower threshold level in FA concentration is reached, UCPs’ proton transport function becomes deactivated, which results in overproduction of ROS species and the cycle is repeated. We observed long-chain FAs, such as OA and AA, to be potent activators of the proton transport activity of neuronal UCPs (Figure 4). These FAs are abundantly found in the CNS and could play a potential role in regulating the function of UCPs in these tissues [53]. The variation in potencies of FAs for activating proton transport in each UCP could imply the existence of distinct and specific hydrophobic FA-binding domains in each protein. As discussed previously, AA was the most potent proton transport activator for UCP2, whereas OA was the most potent proton transport activator for UCP4 and UCP5 (Figure 4; Table 3). In addition, UCP2 transported chloride ∼2-fold faster than UCPs 4 and 5 (Figure 5; Table 3). The difference between UCP2 compared with UCP4 and UCP5 is also reflected in their sequence homology and tissue expression profiles (Table 3). UCP2 is not only found in CNS tissues but is also expressed in liver, pancreas, muscle and other human tissues (Table 3). On this basis, the specific FA activation of UCPs’ proton transport could be linked to the tissue in which each protein is expressed and the availability of FAs in those tissues. Analysis of the FA components of the brain mitochondrial lipids reveals a higher content of OA over AA and DHA (53). Therefore, based on the relative availability of FAs in the brain tissues, it can be speculated that in these tissues, UCP4 and UCP5 are more specifically activated (and, therefore, more capable in transporting protons) than UCP2.

**Figure 7:**
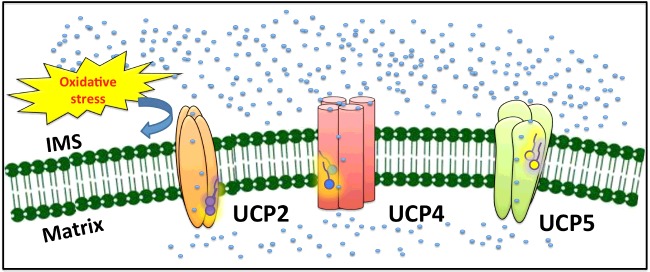
Proposed model of stress-induced proton transport mechanism of neuronal UCPs Under oxidative stress, lipolysis can be activated through enzymes such as mitochondrial phospholipase A2. The lipolysis of mitochondrial phospholipid releases free FAs and their oxidized derivatives (e.g., FAOOH), which can directly interact with UCPs and activate the proton transport. Each neuronal UCP might possess a specific FA-binding site. The FA-binding sites of neuronal UCPs could have an optimal binding affinity for FAs that are available in specific expression tissues. The proton transport mediated by neuronal UCPs can help reduce the membrane potential across the IMM and consequently lower ROS production.

Among neuronal UCPs, UCP4 transported protons and chloride at the lowest rates (Figures 4 and 5). In addition, UCP4 is the only UCP whose ion transport activities were unaffected by CL, a mitochondria-specific lipid that is localized at the cristae membrane of the IMM [11]. These unique biophysical properties of UCP4 could be related to its location within the IMM and its physiological roles. A recent microscopy study revealed that UCP4 is preferentially localized in the IMM inner boundary membrane section, whereas ATP synthase is more centrally located in the cristae membrane section [17]. This local separation of proteins precludes a significant role for UCP4 in uncoupling phosphorylation from proton pumping in the ETC (electron transport chain), but is not in conflict with the original view about the role of UCP4 in modulating excessive proton gradient that is associated with ROS production [17].

In addition to a proton transport-mediated uncoupling effect, UCPs in neurons may indirectly achieve their protective roles. In a recent study, a carrier/exchanger function has been proposed for UCP2 in transporting C4 metabolites out of the mitochondria in exchange for phosphate and proton ions, thereby regulating glucose and glutamine oxidation and, indirectly, reducing ROS production [20]. It is therefore possible that UCP2 possesses different functional modes (i.e. ion transporter and C4 substrate carrier/exchanger) under different physiological conditions. In analogy, despite common structural and functional features shared among neuronal UCPs, UCP4 and UCP5 could also possess other functional modes in addition to their ion transport activities.

## Abbreviations

AA: arachidonic acid
AAC: ADP/ATP carrier
BAT: brown adipose tissue
CMC: critical micelle concentration
CNS: central nervous system
CL: cardiolipin
C_8_E_4_: octyltetraoxyethylene
DHA: docohexanenoic acid
FA: fatty acid
IMM: inner mitochondrial membrane
IMS: intermembrane space
LA: lauric acid
LDAO: lauryldimethylamine-oxide
MCF: mitochondrial carrier family
OA: oleic acid
OG: octyl glucoside
PA: palmitic acid
PC: phosphatidylcholine
PN: purine nt
ROS: reactive oxygen species
POPC: 1-palmitoyl-2-oleoyl-sn-glycero-3-phosphocholine
SPQ: 6-methoxy-N-(3-sulfopropyl)quinolinium
TEA: tetraethylammonium
TEV: tobacco etch virus
TM: transmembrane
UCP: uncoupling protein
